# Some Practical Observations on the Management of Albuminuria in Pregnancy

**Published:** 1891-08

**Authors:** J. F. Y. Paine

**Affiliations:** Galveston


### SOME PRACTICAL OBSERVATIONS ON THE MANAGEMENT OF ALBUMINURIA IN PREGNANCY.

THE following summary of the management of Albuminuria in pregnancy is extracted from an able and exhaustive paper by Prof. Paine, read at the Waco meeting, Texas State Medical Association, April 28, 1891.

"Pregnancy being admitted by universal consent to be a predisposing cause of albuminuria, which is the expression of nephritis and consequent renal insufficiency, the indications of treatment appear clear. Much, however, can be done to counteract this predisposition and maintain a fair standard of general health during the progress of gestation. When a woman becomes pregnant she should be made acquainted with the dangers of her condition. She will doubtless be told that pregnancy is a physiological state; so is digestion; and yet more people die from the effects of indiscretions of diet than from all other causes combined. She should be instructed how to conduct herself that nature's methods may not be contravened. The supplementary functions of skin, lungs bowels and kidneys should be explained to her, and the modes of promoting their activity made clear. The dietary should be so regulated as to meet the requirements of nutrition and preserve a soluble condition of the bowels. The importance of frequent baths and appropriate clothing in connection with the cutaneous secretion can not be too forcibly urged. Her clothing must be loose, to avoid compression of the

---

[J. F. Y. Paine, M. D., Prof. of Obstetrics and Gynecology Med. Dept. University of Texas, in Transactions Texas State Medical Association.]

abdominal organs, and permit free action of lungs and heart. Thin shoes and slippers are never to be worn; they lead to cold feet, contraction of vessels of the lower limbs and ultimate complications. Exercise is essential, it gives bodily strength and promotes elimination. Congenial occupations and innocent recreations keep in the background the mischievous imagination, and conduce to a healthier condition of the nervous system. Social excesses and exciting amusements, as well as strong moral emotions, are to be strictly interdicted. Rest, and an ample amount of sleep, are absolutely required for the maintenance of nervous equilibrium. These details have a general application to the pregnant state, and, if they were observed, would undoubtedly materially diminish the proportion of albuminurics and elevate that condition to a much higher physiological plane.

"When albuminuria is existent, the hygienic expedients enumerated are to be supplemented by medical remedies, and the utmost harmony of opinion prevails among authors and practitioners of experience relative to the therapeutic resources to be employed. The methods of medical treatment consist of non-irritant diuretics, diaphoretics, cathartics which produce large watery stools without much irritation, tonics and nervous sedatives. Hot water and vapor baths, and the wet pack, are most to be relied upon to promote diaphoresis; the weight of evidence being against the use of jaborandi and its alkaloid, on account of their depressing effects upon the heart. One point must be kept in mind, the disease is one of debility, and implies impoverishment of the blood, a condition which calls for tonics and generous diet. Whilst generous, the diet should be simple, unstimulating and easily assimilable, of which milk ought to form a considerable part. Tincture of the chloride of iron is a valuable remedy, it combines diuretic and haematinic properties, and exercises a tonic effect upon weakened vessels. Veratrum viride has been employed during the last few years in cases where the centric nervous system gave evidence of being seriously disturbed, with the effect of averting eclampsic explosion, and improving the general condition.

"The claims of venesection are being urged in high quarters, and clinical evidences favorable to its employment in appropriate cases, seem to be increasing. In the *London Medical Record* of recent date, Dr. V. G. Favr reports the favorable action of the inhalation of oxygen in two cases of albuminuria with serious nervous disturbance; his explanation of its action is, the influence it exercises in controlling morbid reflex irritability.

"By a judicious combination and skillful application of these varied remedial measures, serious issues may commonly be averted and the pregnancy protracted to the term of gestation, or at least until the fœtus is viable. Not unfrequently, however, all efforts are futile, and the albuminuria steadily increases, hydræmia becomes more apparent, anasarca extends, and alarming cerebral, cardiac or pulmonary symptoms follow. In the face of such an extremity, confronted by an imminent danger of great gravity, when conservative methods, which for awhile afforded relief, have all failed, there can be no question about the indication of more active treatment, and the artificial interruption of pregnancy comes up for consideration. Writers on this subject differ widely in their views. Some are unqualifidely opposed to the induction of either abortion or premature labor; others advise accelerative measures only when labor has already begun. The weight of authority seems to favor procrastination, regarding the operation as an extreme measure, justifiable only in cases of utmost peril. With the indications thus limited, premature labor is not likely to save many lives. When shall pregnancy be artificially interrupted? Lusk, Playfair, Braun, Simon, Thomas, Litzman, Hofmeier, Barker and others declare in most unequivocal terms that the induction of premature labor is indicated and justifiable in all cases, in which the quantity of albumen is considerable and progressively increasing in spite of appropriate treatment, and are attended with threatening symptoms, such as persistent headache, sleeplessness and confusion of vision.

"Playfair states that 'the risks of the operation are infinitesimal compared to those which the patient would run in the event of puerperal convulsions supervening, or chronic Bright's disease becoming established.'

"Braun says he 'has known but one patient with eclampsia to recover between the fourth and six months of pregnancy, except where abortion had taken place.'

"My own experience, so far as it goes, coincides with that of Professor Lusk, who asserts that the 'practice of waiting upon nature has proved uniformly disastrous, while the induction of labor has furnished me with a certain proportion of recoveries.'

"Albuminuria strongly predisposes to abortion. According to Braun, one-fourth of albuminurics miscarry; and Tanner states that four out of seven of such cases coming under his observation abort. But evacuation of the uterine contents being the only means of effecting a radical cure of the disorder, its spontaneous occurrence is an event to be desired by obstetricians, and its artificial accomplishment would seem to be an imitation of nature's mode. Without entering into a further discussion of the arguments pertinent to this procedure, I will simply state that clinical experience furnishes incontrovertible evidence in favor of the operation under the limitations designated."

